# Acute-on-Chronic Liver Failure in Severe Alcohol-Associated Hepatitis: Impact on Management, Prognostication, and Urgency of Liver Transplantation

**DOI:** 10.3390/jcm15187020

**Published:** 2026-09-10

**Authors:** Giovanni Perricone, Raffaella Viganò, Chiara Mazzarelli, Chiara Becchetti, Giulia Dispinzieri, Adelaide Panariello, Paola Prandoni, Sara Conti, Paolo Angelo Cortesi, Mario Camozzi, Gianpaola Monti, Andrea Lauterio, Stefano Di Sandro, Luca Saverio Belli

**Affiliations:** 1 Hepatology and Gastroenterology Unit, ASST Grande Ospedale Metropolitano Niguarda, Piazza Ospedale Maggiore 3, 20162 Milan, Italy; 2 Mental Diseases Unit, ASST Grande Ospedale Metropolitano Niguarda, Piazza Ospedale Maggiore 3, 20162 Milan, Italy; 3 Occupational Health Service, General Management, University of Milano-Bicocca, 20900 Milan, Italy; 4 Research Centre on Public Health (CESP), University of Milano-Bicocca, 20900 Monza, Italy; 5 Pathology Unit, ASST Grande Ospedale Metropolitano Niguarda, Piazza Ospedale Maggiore 3, 20162 Milan, Italy; 6 Transplant Intensive Care Unit, ASST Grande Ospedale Metropolitano Niguarda, Piazza Ospedale Maggiore 3, 20162 Milan, Italy; 7 General Surgery and Transplantation Unit, ASST Grande Ospedale Metropolitano Niguarda, Piazza Ospedale Maggiore 3, 20162 Milan, Italy

**Keywords:** severe acute alcohol-associated hepatitis, acute-on-chronic liver failure, death, liver transplantation, prognostication

## Abstract

**Background/Objectives:** Severe alcohol-associated hepatitis (SAH) is a complication of alcoholic liver disease associated with high mortality. Effective identification of factors predicting the course, outcome, and need for early liver transplantation (eLT) in patients with SAH is crucial. Acute-on-chronic liver failure (ACLF), a syndrome characterized by an acute decompensation of cirrhosis, failure of one or more organs, and high short-term mortality, can occur in SAH, and its impact on management, prognostication, and need for eLT should be clarified. **Methods:** This is a single-center, retrospective study of patients with SAH admitted to the ASST GOM Niguarda in Milan between April 2016 and May 2023. **Results:** One hundred patients were included. Median Maddrey’s discriminant function (MDF) was 72.1 (IQR 51.6–101.3), and the median model for end-stage liver disease—sodium (MELD-Na) score was 28.5 (IQR 25.0–32.5). At presentation, 57 patients (57%) had ACLF 0–1 (54 ACLF 0—no organ failure [OF] or single non-renal OF with serum creatinine < 1.5 mg/dl and no hepatic encephalopathy; 3 ACLF 1—defined by the presence of at least renal failure or any other single OF if associated with renal dysfunction and/or grade I–II hepatic encephalopathy), and 43 patients (43%) had ACLF 2–3 (31 ACLF 2—defined by the presence of two OFs, 12 ACLF 3—defined by the presence of three or more OFs). In the latter group, circulatory failure was present in two (4.65%) and respiratory failure in three (6.98%). SAH was the only precipitating factor in 3 (100%) and 39 (90.7%) patients with ACLF 1 and ACLF 2–3, respectively, with infection being an associated factor in 5 (11.63%) and GI bleeding in 2 (4.65%) of subjects with ACLF 2–3. Non-response to medical treatment (MT) was significantly higher in patients with ACLF 2–3 (38/43, 88.4%) than in those with ACLF 0–1 (25/57, 43.8%). ACLF 2–3 was associated with a corresponding higher need for eLT (ACLF 2–3: 15/43, 34.9% vs. ACLF 0–1: 10/57, 17.54%%; *p* = 0.0474). ACLF status at presentation (ACLF 2–3 vs. ACLF 0–1) has a similar predictive accuracy of outcome (death or liver transplantation [LT]) to MELD-Na (as a 1-unit increase) (3.261 [1.999; 5.318], concordance index 0.702 vs. 1.154 [1.094; 1.217], concordance index 0.708). **Conclusions:** ACLF 2–3 is a frequent presentation of SAH, 43% of the cases. Given that most of these patients do not respond to MT (approximately 90%), fast-track evaluation for LT is advised for patients with a favorable psycho-social profile.

## 1. Introduction

Alcohol-associated hepatitis (AH) is a clinical syndrome occurring in patients with chronic and active heavy alcohol use, which typically presents with an abrupt onset of jaundice, malaise, decompensated liver disease, and coagulopathy. AH is diagnosed according to the criteria proposed by the National Institute on Alcohol Abuse and Alcoholism (NIAAA): last alcoholic drink within 8 weeks of onset of jaundice; serum bilirubin level > 3 mg/dL; AST and ALT level < 400 IU/L and AST level > 50 IU/mL, with the ratio of AST to ALT > 1.5:1; and other causes of liver disease, biliary obstruction, and hepatocellular carcinoma ruled out. Liver biopsy may be required for patients with an uncertain clinical diagnosis or according to center practice. In its more severe form, identified by Maddrey’s discriminant function (MDF) ≥ 32 or the model for end-stage liver disease (MELD) score > 20, the use of corticosteroids (CSs) represents an important component of the medical treatment (MT) because it is potentially effective in reducing the short-term mortality risk [1,2,3,4].

Acute-on-chronic liver failure (ACLF) is now an established syndrome that can complicate the course of chronic liver disease including alcohol-related liver disease. Various definitions have been proposed by major societies across the globe; the Kyoto consensus has recently redefined ACLF to be inclusive of all global definitions [5]. According to the European Association for the Study of the Liver (EASL)-Chronic Liver Failure (CLIF) Consortium, ACLF is characterized by acute decompensation (AD) (ascites, hepatic encephalopathy, bacterial infection, bleeding), organ failures (OFs), and a high short-term mortality [6], with heavy alcohol consumption being one of the main triggers of the syndrome [7,8,9]. The study by Sersté et al. showed that ACLF is frequent either at presentation or during the course of severe AH (SAH), is characterized by poor response to CSs, particularly in patients with ACLF 2–3, and is associated with high mortality [3].

To the best of our knowledge, the role of ACLF in patients with SAH evaluated for liver transplantation (LT) is largely understudied. Therefore, we aimed to address the following issues: (a) natural history of ACLF as a presenting or complicating syndrome of SAH; (b) whether the presence of ACLF affects the response to MT; and (c) the impact of ACLF on prognosis and urgency for LT.

## 2. Patients and Methods

### 2.1. Study Population

This is a single-center cohort study of all consecutive patients admitted for SAH to the ASST GOM Niguarda, Milan, Italy (retrospective analysis of a prospective database). All patients signed a general consent form of the hospital allowing reuse of their health-related data. The present study analyzed data for patients admitted between April 2016 and May 2023.

This study was approved by the local institutional review board (EC Milano Area 3, code 4814, date 12 June 2023) and was conducted in agreement with the local legislation and the Declaration of Helsinki.

### 2.2. Assessment of SAH

The diagnosis of AH was based on clinical history, physical examination, and laboratory findings according to the NIAAA criteria; liver biopsy through a transjugular route was done in patients with confounding factors [10]. For patients undergoing LT, histology of the native liver was reviewed to confirm the diagnosis of AH. Baseline parameters were collected at the time of the index episode of SAH. The severity of AH was assessed using the MDF and the MELD and model for end-stage liver disease—sodium (MELD-Na) scores. Inactive hepatitis B or C infection or with hepatocellular carcinoma did not represent an exclusion criterion.

### 2.3. Assessment of ACLF

The criteria for ACLF diagnosis and grades were those of the European Association for the Study of the Liver (EASL)-Chronic Liver Failure (CLIF) Consortium [11], which were derived from the CANONIC study [6]. Briefly, ACLF was defined as a syndrome characterized by acute decompensation (acute development of ascites, hemorrhage, encephalopathy, and/or bacterial infections) of cirrhosis, associated with OFs (liver, kidney, brain, coagulation, circulation, and/or respiration) and a high short-term mortality rate (defined as a mortality rate equal to or greater than 15% within a period of 28 days). The diagnosis of ACLF was based on the presence of at least renal failure or any other single organ failure if associated with renal dysfunction (serum creatinine 1.5–1.9 mg/dl) and/or grade I–II hepatic encephalopathy (ACLF-1). Patients with two organ failures were graded as ACLF-2 and those with three or more organ failures as ACLF-3.

### 2.4. Treatment Protocol for SAH and Candidate Selection for LT

The cornerstones of MT were alcohol abstinence, enteral nutrition for caloric and protein intake (35–40 kcal/kg per day, with protein 1.5 g/kg per day), early identification and appropriate treatment of infections, prevention and appropriate treatment of organ dysfunction or failure, treatment of the complications of portal hypertension, and the use of CSs (prednisolone or prednisone 40 mg per day orally or methylprednisolone 32 mg per day intravenously) in selected patients [12]. Active uncontrolled infection, ACLF 3 and/or MELD score > 30, were considered contraindications for CSs. In addition, patients who improved spontaneously before being transferred to our unit did not receive CSs.

Exclusion criteria for LT were based on those used in the study by Mathurin et al. [13], with the following exceptions: patients with controlled psychiatric disorders, patients with underlying cirrhosis, and patients with a prior episode of AH or liver decompensation, in case they had never been evaluated and supported by a mental health specialist in the past, were evaluated for LT in our center.

A dedicated psychiatrist and psychologist evaluated all LT candidates in conjunction with the caregiver, in general a family member and a social assistant whenever needed, as previously described [14]. Patients were considered not eligible for LT if one or more of the following conditions were present: poor awareness of alcohol use disorder and lack of willingness to abstain; unsatisfactory Global Assessment of Functioning (GAF < 70); psychiatric disturbances that were not deemed treatable; presence of significant cognitive impairment as assessed by neuropsychological tests prescribed by a dedicated psychiatrist on clinical suspicion; inadequate social support and housing conditions; ongoing substance use disorder other than cannabis and methadone. The presence of at least 1 of the aforementioned conditions identified a patient with an unfavorable addiction/social profile.

### 2.5. Definitions

#### 2.5.1. Response to MT

Response to MT was defined as clinical improvement and was based on the Lille model (<0.45 at 7 days) or on bilirubin trajectory (bilirubin < 0.8 × admission value at 7 days), reflecting an early favorable trajectory of liver disease. Conversely, non-response to MT was based on the Lille model or on an increase in the MELD and/or MELD-Na score [15,16,17].

#### 2.5.2. Early vs. Standard LT

We adhered to the definition of the ACCELERATE study [18], which defines the window for early LT (eLT) related to AH within 6 months of the index episode of SAH. eLT was considered for patients non-responding to MT and with rapidly worsening liver function [13,18,19,20,21,22,23].

#### 2.5.3. Prevalent vs. Incident ACLF

ACLF can be established at the time of diagnosis of AH (prevalent ACLF [pACLF]) but can also develop during the course of medical management of this disease (incidental ACLF [iACLF]) [3].

## 3. Statistical Analysis

A descriptive analysis of the cohort was carried out on the overall population and following stratification of the population by ACLF severity (no ACLF/pACLF 1 vs. pACLF 2–3). Continuous and categorical variables were summarized using absolute and relative frequencies and the median and interquartile range, respectively. Continuous group characteristics were compared using the Wilcoxon signed-rank test, while categorical ones were compared using Fisher’s exact test. The association between the occurrence of death or LT, as a composite outcome, and baseline clinically relevant characteristics (age, sex, MELD, MELD-Na, and ACLF grade) was evaluated through multivariate Cox proportional hazard models. The predictive accuracy of such models was compared using Harrell’s C-index (concordance index) [24]. Survival before transplant and the probability of death or transplant were estimated using the Kaplan–Meier method and compared between no ACLF/pACLF 1 and pACLF 2–3 using the log-rank test. A focus on non-responders to MT was carried out by estimating Kaplan–Meier curves of the probability of transplant and of the overall survival probability, stratified by ACLF severity at baseline (no ACLF/pACLF 1 vs. pACLF 2–3). Time was measured from the first day of hospitalization to the last known date of follow-up or date of death from any cause. All statistical analyses were conducted using R V.4.4.2 (R Core Team, Vienna, Austria).

## 4. Results

During the study period, 100 consecutive patients with SAH were included; 63 (63%) were male, median (IQR) age was 50.5 years (44–56), 95 (95%) were white, and 70 (70%) had been referred from other hospitals. Prior liver decompensation (AH or complications due to clinically significant portal hypertension) was present in 29 (29%) patients. Other clinical characteristics, biochemical values, and treatment at inclusion are summarized in Table 1. Liver biopsy confirmation of alcohol-related hepatitis was available in the 30 patients with clinical AH classified as possible AH according to NIAAA criteria.

**Figure 1:**
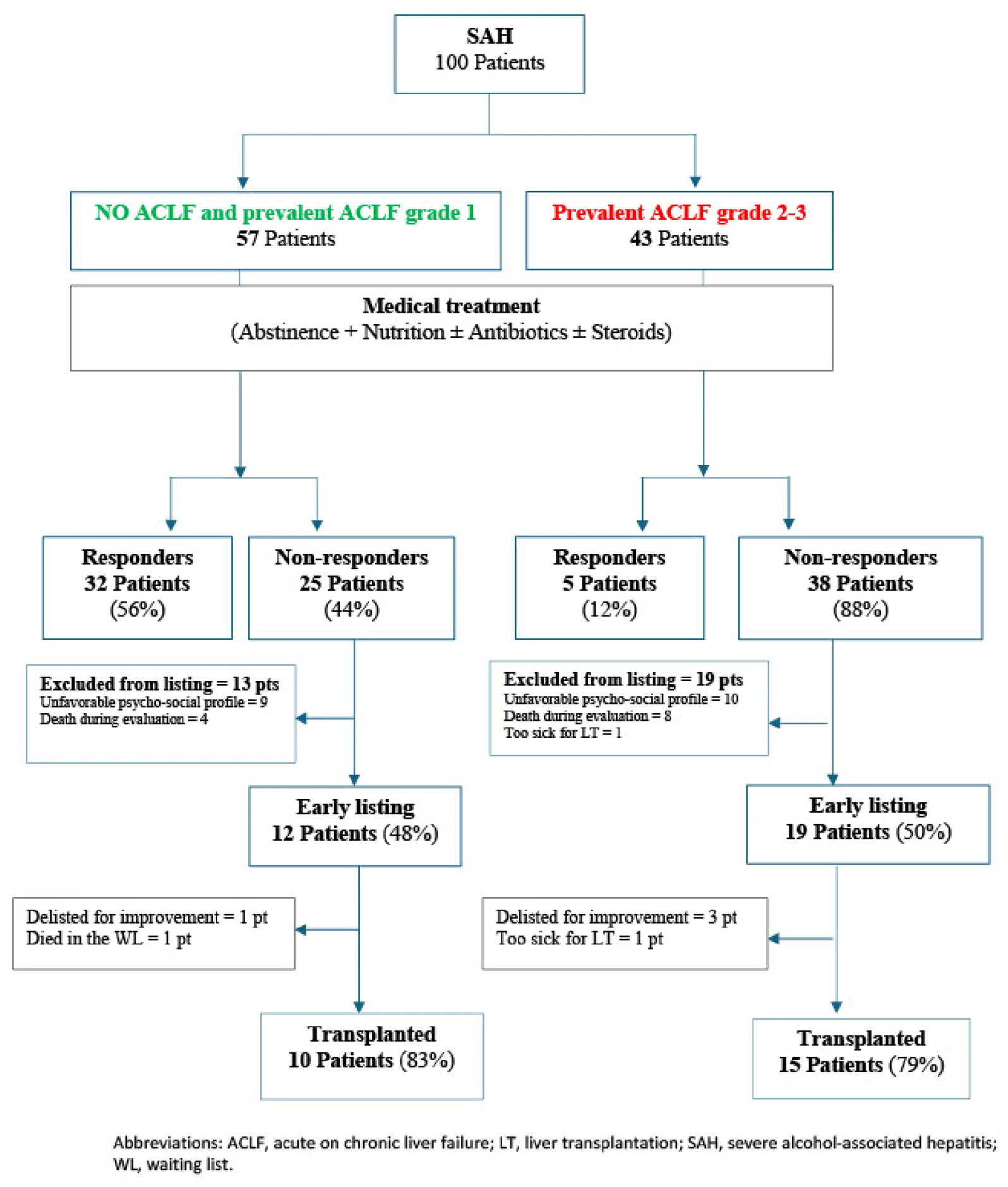
Patient disposition.

Overall, 37 patients (37%) were considered responders to MT (Figure 1). Twenty-five patients (25%) received CSs. Among patients not using CSs, the main reasons for not using them were spontaneous improvement in 25 patients (33.3%), confirmed or presumed uncontrolled infection in 17 patients (22.7%), pACLF 2–3 in 28 patients (37.3%), and other reasons at the discretion of the medical team in 5 patients (6.7%). Notably, 30 patients (30%) were referred to our center > 2 weeks after the index episode of SAH at the local hospital.

At admission, median (IQR) MDF was 72.1 (51.6–101.3), and the median (IQR) MELD-Na score was 28.5 (25–32.5).

### 4.1. Characteristics of ACLF

#### 4.1.1. Prevalent ACLF

Overall, 46 patients had ACLF already at admission, pACLF. Three patients (3%) had pACLF 1, 31 patients (31%) had pACLF 2, and 12 patients (12%) had pACLF 3. There were no differences in the events defining AD between no ACLF/pACLF 1 and pACLF 2–3, with ascites being the most frequent (80%).

pACLF 1 group. In this small group of three patients, SAH was the precipitating factor in all three cases (100%). Liver failure was associated with cerebral dysfunction in two patients and renal dysfunction in one patient (Table 1).

pACLF 2–3 group. This group consisted of 43 patients, where SAH was the precipitating factor in all cases, with infection and GI bleeding being associated cofactors in five (11.6%) and two patients (4.65%), respectively. Liver failure was the most frequent OF (39 patients, 90.7%), followed by coagulation failure (31 patients, 72.09%), renal failure (18 patients, 41.86%), and brain failure (8 patients, 18.6%); circulatory failure was present in two patients (4.65%) and respiratory failure in three patients (6.98%) (Table 1).

#### 4.1.2. Incidental ACLF

Fifty-four patients did not have pACLF. Among these, seven cases of iACLF were recorded. The precipitating factor was an infection in five patients and variceal bleeding in two patients. Two patients had ACLF 1b (liver failure and cerebral dysfunction in both cases), one patient ACLF 2 (liver and renal failures), and four patients ACLF 3 (liver, cerebral, and respiratory failures in one case; liver, coagulation, renal, and cerebral failures in one case; liver, coagulation, and renal failures in one case; liver, coagulation, and circulatory failures in one case).

### 4.2. Infections

Fifty-three patients (53%) developed an infection without significant differences between no ACLF/pACLF 1 and pACLF 2–3. Bloodstream infections, pneumonia, and urinary tract infections were the most frequent sites. The most frequent organisms were fungi, followed by Gram-negative extended-spectrum beta-lactamase (ESBL)-positive bacteria and vancomycin-sensitive/resistant Enterococcus (VSE/VRE) (Table 2).

### 4.3. Impact of ACLF Grades on MT Response

Non-response to MT was significantly higher in patients with pACLF 2–3 (38/43 patients, 88.4%) than in those with no ACLF/pACLF 1 (25/57 patients, 43.8%), *p* < 0.0001 (Figure 1, Table 1).

### 4.4. Impact of ACLF on Prognosis and Urgency for LT

pACLF 2–3 was associated with a corresponding higher need for eLT (pACLF 2–3 15/43 patients, 34.9%, vs. no ACLF/pACLF 1 10/57 patients, 17.5%; *p* = 0.047) (Table 1). Nineteen out of twenty patients with pACLF 2–3 non-responders to MT listed for eLT had a MELD-Na score ≥ 30.

**Figure 2:**
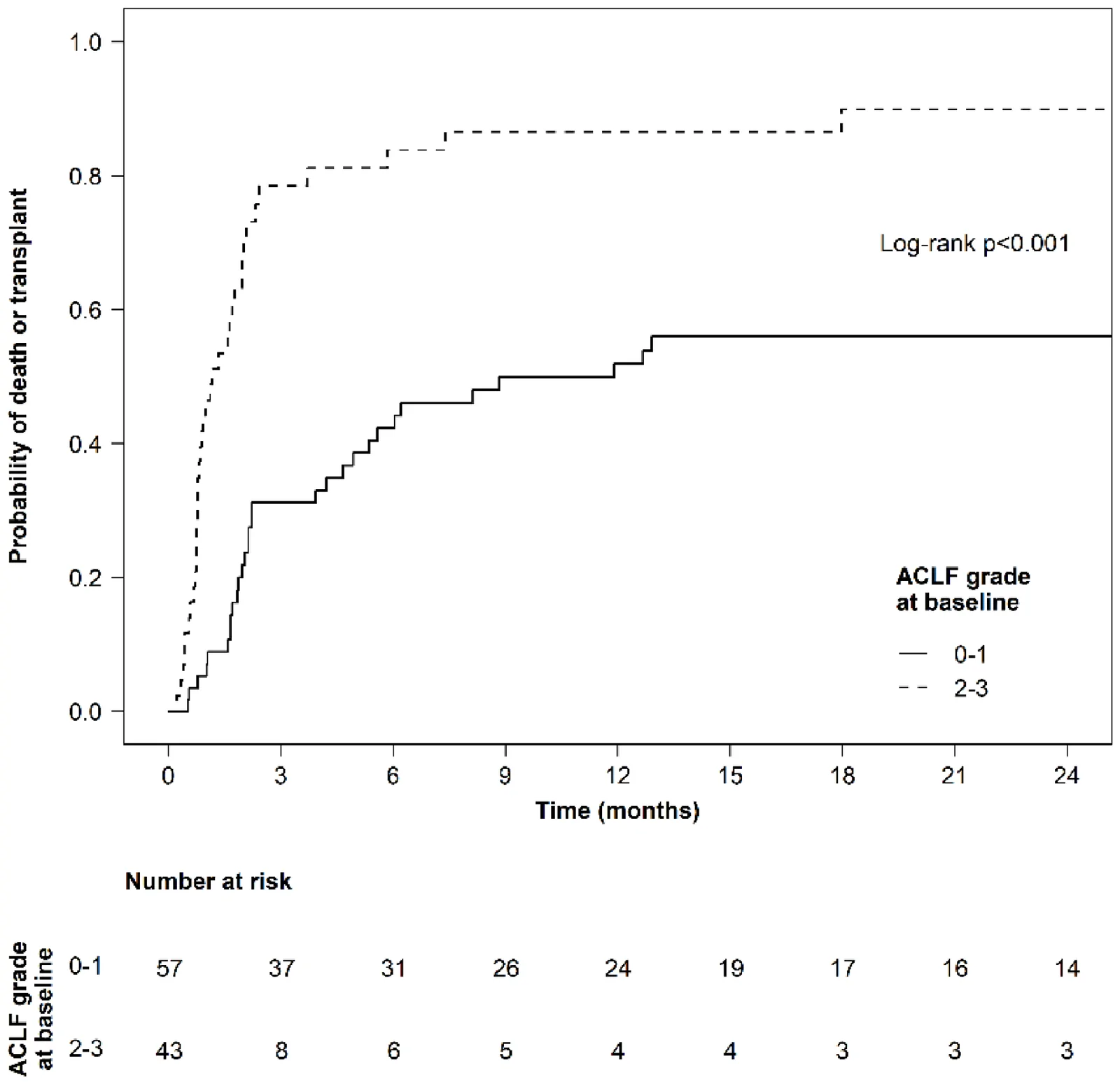
Kaplan–Meier estimates of the probability of death or transplant, from the day of index admission for SAH, stratified for NO ACLF/pACLF 1 (solid line) and prevalent pACLF 2–3 (dotted line).

The Kaplan–Meier estimates of the probability of death or transplant from the day of index admission for SAH, stratified for NO ACLF/pACLF 1 and pACLF 2–3, are given in Figure 2, where patients with pACLF 2–3 were exposed to a significantly higher risk of death or LT.

**Figure 3:**
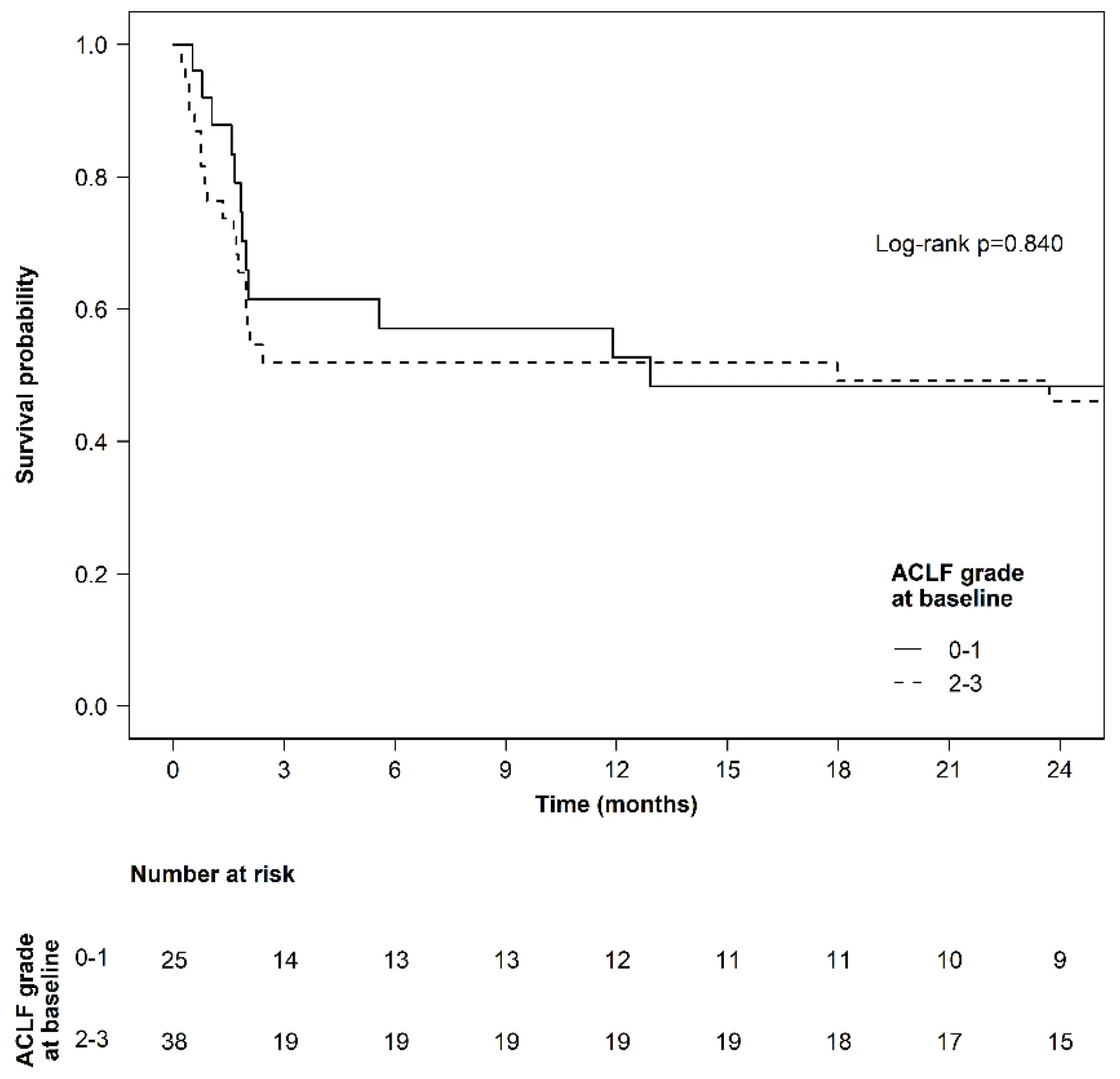
Kaplan–Meier estimates of the survival probability among non-responders to medical treatment, from the day of index admission for SAH, stratified for no ACLF/prevalent ACLF 1 (solid line) and prevalent pACLF 2–3 (dotted line).

The Kaplan–Meier estimates of the survival probability among non-responders to MT from the day of index admission for SAH, stratified for no ACLF/pACLF 1 and pACLF 2–3, are given in Figure 3, where the probability of survival was similar between the two groups.

### 4.5. Predictors of Death or Liver Transplantation

According to multivariate models including age, sex, MELD-Na score, ACLF grade, and CLIF-C ACLF score, both MELD-Na score and the presence of pACLF 2–3 at index hospitalization were associated with the probability of death or LT (Table 3). The increase of 1 unit in MELD-Na score was associated with an HR of 1.154 (95% CI: 1.094–1.217) of death or LT; patients with MELD-Na ≥ 30 were 2.416 (95% CI: 1.452–4.019) times more likely to die or be transplanted than those with a MELD-Na score < 30. Patients with pACLF-2–3 were 3.261 (95% CI: 1.999–5.318) times more likely to die or be transplanted compared to those with no ACLF/pACLF 1. The concordance index (higher values indicate a better model fit) was similar for the models including MELD-Na score (as 1 unit increase) and the presence of severe pACLF, suggesting a similar predictive accuracy of these two variables.

## 5. Discussion

To the best of our knowledge, this is one of the few studies exploring the natural history of ACLF complicating the clinical presentation of SAH, including its impact on response to MT [3], and it is the first to explore prognosis and urgency of LT in this specific setting.

This work confirms that ACLF is frequent in patients admitted for SAH, with 46% presenting with ACLF of different severity at index hospitalization—3% with ACLF 1, 31% with ACLF 2, and 12% with ACLF 3—with SAH being the only precipitant in most of the cases. These data are in line with the findings of the study by Sersté et al. [3], where the prevalence of ACLF in their patients with biopsy-proven SAH was 47.9%.

Agreement exists that the MT of patients with SAH should be based on alcohol abstinence, nutrition, early identification and appropriate treatment of infections, and the use of CSs in eligible patients [2]. Sersté et al. showed that the rates of response to CSs were much lower in patients with ACLF, being 42.1% for patients with pACLF 2 and only 8.3% for those with pACLF 3, compared to 76.6% and 52.2% for patients without ACLF and those with pACLF 1, respectively [3]. These findings were also confirmed in a sub-analysis of the STOPAH trials, where the proportion of Lille responders was low in patients with ACLF 2–3 [25], being 37.5% in patients treated and 34% in those untreated with CSs, again putting into doubt the utility of CSs in the management of SAH presenting with ACLF [26]. Based on these data, a very recent statement from EASL guidelines warns that with increasing severity of ACLF, responsiveness to CSs is progressively reduced while the risk of infection increases and specifically recommends against CS use in ACLF 3 [27]. In the current study, only 25% of the patients in the whole cohort received CSs, a percentage which is lower than that reported in the ACCELERATE real-life study from the US (54%) [18] and in the Italian multicenter study (48%) [22]. This is explained by our approach not to start CSs in patients who showed a spontaneous clinical improvement before being referred to our center, also considering that 30% were transferred > 2 weeks after the index episode of SAH at the local hospital, and by the policy adopted since 2018 of a very cautious use of CSs in patients with ACLF 2–3. Not only were we influenced by the data provided by Sersté et al. [3], but we were also concerned that the use of CSs in patients without contraindication for LT would add to post-LT immunosuppression, thus resulting in a state of over-immunosuppression and a higher risk for severe infectious complications early post-LT. This worry was recently reported by Lee and coll. [18] who observed that post-LT mortality, attributable to severe infections, was significantly higher in patients who had received CSs prior to LT. Concurrently, in the seminal study by Mathurin and colleagues [13], the occurrence of invasive infections was the leading cause of early death following LT. Similarly, in a recent paper by Mohy-Ud-Din et al., the authors propose to defer CSs in the most severe subset of SAH, defined as a MELD score ≥ 35 or MDF ≥100 and termed “catastrophic” SAH, and to expedite eLT evaluation during the index admission [28]. All these more recent studies are for a cautious use of CSs in patients with ACLF 2–3 who are potentially eligible for LT, considering that the impact of CSs on survival is small and temporary, and the risk of post-LT infection is high.

Regarding the issue of which impact ACLF has on prognosis and urgency for LT, this study confirmed that ACLF predicts poor response to MT, this being significantly higher in patients with pACLF 2–3 (88.4%) than in those with no ACLF/pACLF 1 (43.8%). Therefore, patients with pACLF 2–3 without contraindications for LT should be rapidly assessed for listing, considering the high 30-day mortality risk without transplant. An open debate is ongoing regarding the prioritization beyond the MELD-Na score for patients with ACLF, with some countries offering priority to patients with ACLF 3 [29,30,31]. This work showed that the ACLF grade and the CLIF-C ACLF score were not superior to the MELD-Na score for predicting death or eLT, which is not surprising given the fact that most patients with ACLF 2–3 had a different combination of liver failure (91%), coagulation failure (72%), and renal failure (42%) which are well captured by the MELD-Na score, and only a minority had respiratory failure (7%), circulatory failure (5%), or brain failure (19%), which are not considered in the MELD-Na score. As a matter of fact, 19 out of 20 patients listed with ACLF in this cohort had a MELD-Na score higher than 30, which allowed adequate macroregional priority in Italy (median waiting time of 2 days between listing and LT). Conversely, the issue of inadequate prioritization arises in the case of ACLF 2–3 patients with low MELD scores, this finding being clearly highlighted in studies based on the UNOS database, where ACLF 3 patients with respiratory, circulatory, and brain organ failures are highly prevalent [30,32]. In addition, it is important to underline that the CLIF-C ACLF score was not designed to predict mortality in patients with SAH, so its interpretation should be integrated with other scores including MELD-Na score and MDF.

A final encouraging finding of this work is that the estimates of the survival probability among non-responders to MT are similar between no ACLF/pACLF 1 and pACLF 2–3. This finding is in contrast with the results of the ECLIS study [33], where 30% of ACLF 2–3 patients died on the WL. We believe that our short waiting time after listing (median of only 2 days) was crucial to achieve this goal.

We acknowledge some limitations. First, the relatively small number of patients did not allow for more granular analysis. In particular, the role of sarcopenia could not be adequately explored, as this information was available only for 70% of the cases, with many patients with a rapid negative trajectory at the time of admission not surviving long enough to have a CT scan done. Second, the cautious use of CSs was set a priori while only a randomized controlled trial including patients with SAH and ACLF 2–3 with and without CSs would make it possible to assess differences in their responses and their survival.

## 6. Conclusions

ACLF is a frequent presentation of SAH, in 46% of the cases. As the vast majority do not respond to MT (approximately 90%), fast-track evaluation for LT is advisable, specifically in those without medical contraindications and with a favorable psycho-social profile. At least in Italy, the MELD-Na score was adequate to ensure the right priority in patients listed with ACLF.

## Figures and Tables

**Table 1:** Characteristics of patients according to prevalent ACLF 2–3 or NO ACLF/prevalent ACLF 1 at admission for SAH.

	NO ACLF/pACLF 1	pACLF 2–3	Overall	*p*-Value
	(N = 57)	(N = 43)	(N = 100)	
**Sex male,** n (%)	35 (61.40%)	28 (65.12%)	63 (63.00%)	0.7034
**Age,** years, median (IQR)	50.0 (43.0–55.0)	52.0 (45.0–56.0)	50.5 (44.0–56.0)	0.3139
**Referred from other hospital**, n (%)	34 (59.65%)	36 (83.72%)	70 (70.00%)	0.0093
**First episode of decompensation,** n (%)	43 (75.44%)	28 (65.12%)	71 (71.00%)	0.2601
**Treated with CS,** n (%)				0.7264
Yes	15 (26.32%)	10 (23.26%)	25 (25.00%)	
No	42 (73.68%)	33 (76.74%)	75 (75.00%)	
**Non-response to MT**, n (%)	25 (43.86%)	38 (88.37%)	63 (63.00%)	<0.0001
**Reasons for not receiving CS**, n (%) ^a^				<0.0001
ACLF grade 3	1 (2.38%)	16 (48.48%)	17 (22.67%)	
ACLF grade 2	1 (2.38%)	10 (30.30%)	11 (14.67%)	
Presumed or confirmed uncontrolled infection	12 (28.57%)	5 (15.15%)	17 (22.67%)	
Spontaneous improvement	24 (57.14%)	1 (3.03%)	25 (33.33%)	
Other reasons	4 (9.52%)	1 (3.03%)	5 (6.67%)	
**WBC,** cells/mm^3^, median (IQR)	10,500.0 (6400.0–12,760.0)	12,750.0 (8100.0–17,310.0)	11,120.0 (6930.0–15,165.0)	0.0975
**INR,** median (IQR)	1.8 (1.6–2.1)	2.6 (2.0–3.3)	2.0 (1.7–2.6)	<0.0001
**Creatinine,** mg/dL, median (IQR)	0.7 (0.6–0.9)	1.0 (0.7–2.5)	0.8 (0.6–1.2)	0.0006
**Bilirubin,** mg/dL, median (IQR)	15.0 (10.0–22.0)	21.0 (15.0–30.0)	17.4 (11.5–24.8)	0.0093
**Maddrey score day 0**, median (IQR)	56.0 (44.8–73.0)	102.0 (75.6–146.8)	72.1 (51.6–101.3)	<0.0001
**MELD-Na score day 0,** median (IQR)	26.0 (23.7–28.2)	34.0 (29.0–38.4)	28.5 (25.0–32.5)	<0.0001
**Lille score,** median (IQR)	0.5 (0.1–0.8)	0.9 (0.6–1.0)	0.6 (0.3–0.9)	<0.0001
**ACLF at day 0,** n (%)				<0.0001
No ACLF	54 (94.74%)	(0.00%)	54 (54.00%)	
ACLF 1	3 (5.26%)	(0.00%)	3 (3.00%)	
ACLF 2	(0.00%)	31 (72.09%)	31 (31.00%)	
ACLF 3	(0.00%)	12 (27.91%)	12 (12.00%)	
**Acute decompensation**, n (%) ^b^				
Gastrointestinal bleeding	3 (5.26%)	2 (4.65%)	5 (5.00%)	1
Ascites	42 (73.68%)	38 (88.37%)	80 (80.00%)	0.0815
Infection	4 (7.02%)	4 (9.30%)	8 (8.00%)	0.7223
Hepatic encephalopathy	8 (14.04%)	9 (20.93%)	17 (17.00%)	0.3635
**Organ failures** ^c^, n (%)				
Liver	3 (5.26%)	39 (90.70%)	42 (42.00%)	<0.0001
Coagulation	(0.00%)	31 (72.09%)	31 (31.00%)	<0.0001
Renal	(0.00%)	18 (41.86%)	18 (18.00%)	<0.0001
Brain	(0.00%)	8 (18.61%)	8 (8.00%)	0.0008
Circulation	(0.00%)	2 (4.65%)	2 (2.00%)	0.1824
Lung	(0.00%)	3 (6.98%)	3 (3.00%)	0.0763
**Organ dysfunction ^c^**, n (%)				
Cerebral	2 (3.51%)	(0.00%)	2 (2.00%)	0.5048
Renal	1 (1.75%)	(0.00%)	1 (1.00%)	1
**Type of precipitant of ACLF**, n (%) ^d^				
Alcohol (as only precipitant)	3 (100.00%)	39 (90.70%)	42 (91.30%)	1.0000
Associated infection	(0.00%)	5 (11.63%)	5 (10.87%)	1.0000
Associated bleeding	(0.00%)	2 (4.65%)	2 (4.35%)	1.0000
**L3SMI Area (cm^2^/m^2^),** median (IQR)	38.2 (32.3–44.9)	41.1 (34.3–52.6)	39.3 (32.8–46.9)	0.1018
**Sarcopenia** ^^^, n (%) ^e^	35 (61.40%)	18 (41.86%)	53 (53.00%)	0.0687
**Deaths**, n (%)	21 (36.84%)	22 (51.16%)	43 (43.00%)	0.1521
**Time to death**, months, median (IQR)	2.2 (1.8–12.7)	1.1 (0.7–2.0)	1.9 (0.8–5.6)	0.0021
**Transplant**, n (%)	13 (22.81%)	17 (39.53%)	30 (30.00%)	0.0707
Within 6 months (eLT)	10 (17.54%)	15 (34.88%)	25 (25.00%)	0.0474
**Time to LT**, days, median (IQR)	128.0 (52.0–163.0)	30.0 (24.0–71.0)	51.5 (25.0–150.0)	0.0279
**Follow-up time**, months, median (IQR)	20.9 (2.2–43.4)	2.4 (0.9–49.5)	17.7 (1.9–45.7)	0.1062

Notes: ^a^ Percentage refers to patients not receiving CSs. ^b^ More than one event possible. ^c^ More than one OF possible. ^d^ Percentage refers to patients with ACLF. ^e^ Defined as L3SMI Area <50 cm^2^/m^2^ for men and <39 cm^2^/m^2^ for women. ^^^ 30 missing values. Abbreviations: ACLF, acute-on-chronic liver failure; CS, corticosteroid; INR, international normalized ratio; L3SMI, L3 skeletal muscle index; MT, medical treatment; SAH, severe alcohol-associated hepatitis; WBC, white blood cells.

**Table 2:** Prevalence and characteristics of infections according to prevalent ACLF 2–3 or NO ACLF/prevalent ACLF 1 at admission.

	NO ACLF/pACLF 1	pACLF 2–3	Overall	*p*-Value
	(N = 57)	(N = 43)	(N = 100)	
**N of patients with infection**, n (%)	28 (49.12%)	25 (58.14%)	53 (53.00%)	0.3711
**Site**, n (%)				
SBP	6 (10.53%)	3 (6.98%)	9 (9.00%)	0.7284
UTI	7 (12.28%)	6 (13.95%)	13 (13.00%)	0.8055
Pneumonia	7 (12.28%)	9 (20.93%)	16 (16.00%)	0.2428
SSTI	0 (0.00%)	1 (2.33%)	1 (1.00%)	0.43
Bloodstream infection	11 (19.30%)	13 (30.23%)	24 (24.00%)	0.205
CRBSI	2 (3.51%)	4 (9.30%)	6 (6.00%)	0.3979
Secondary bacteremia	4 (7.02%)	5 (11.63%)	9 (9.00%)	0.4929
Spontaneous bacteremia	2 (3.51%)	3 (6.98%)	5 (5.00%)	0.6491
Cholecystitis	2 (3.51%)	0 (0.00%)	2 (2.00%)	0.5048
Colitis	0 (0.00%)	1 (2.33%)	1 (1.00%)	0.43
Unknown site	2 (3.51%)	0 (0.00%)	2 (2.00%)	0.5048
**MDRO/Fungi**, n (%)				
Gram −ve carbapenem-resistant	0 (0.00%)	2 (4.65%)	2 (2.00%)	0.1824
Gram −ve ESBL +ve	3 (5.26%)	2 (4.65%)	5 (5.00%)	1
VRE/VSE	4 (7.02%)	0 (0.00%)	4 (4.00%)	0.1322
MRSA/VRSA	1 (1.75%)	0 (0.00%)	1 (1.00%)	1
Fungi	3 (5.26%)	3 (6.98%)	6 (6.00%)	1
Other	4 (7.02%)	1 (2.33%)	5 (5.00%)	0.3874

Abbreviations: CRBSI, catheter-related bloodstream infection; ESBL, extended-spectrum beta-lactamase; MRSA, methicillin-resistant Staphylococcus aureus; SBP, spontaneous bacterial peritonitis; SSTI, skin and soft tissue infection; UTI, urinary tract infection; VRE, vancomycin-resistant enterococci; VRSA, vancomycin-resistant Staphylococcus aureus; VSE, vancomycin-sensible enterococci.

**Table 3:** Results from Cox proportional hazard models for predicting death or LT.

	Hazard Ratio	*p*-Value	Concordance Index
	(95% Confidence Interval)		
**Model 1**			0.708
Sex (Male vs. Female)	0.902 (0.547–1.487)	0.685	
Age (1 unit increase)	1.033 (1.002–1.064)	0.035	
**MELD-Na score (1 unit increase)**	**1.154 (1.094–1.217)**	**<0.001**	
**Model 2**			0.664
Sex (Male vs. Female)	0.893 (0.532–1.499)	0.669	
Age (1 unit increase)	1.026 (0.996–1.057)	0.091	
**MELD-Na score (≥30 vs. <30)**	**2.416 (1.452–4.019)**	**0.001**	
**Model 3**			0.702
Sex (Male vs. Female)	1.190 (0.727–1.947)	0.490	
Age (1 unit increase)	1.028 (0.997–1.059)	0.075	
**pACLF (2–3 vs. 0–1)**	**3.261 (1.999–5.318)**	**<0.001**	
**Model 4**			0.691
Sex (Male vs. Female)	1.282 (0.782–2.103)	0.325	
**CLIF-C ACLF score (1 unit increase)**	**1.055 (1.033–1.078)**	**<0.001**	

## Data Availability

Dataset available on request from the authors.

## Abbreviations

ACLF, acute-on-chronic liver failure; AH, alcohol-associated hepatitis; CLIF, chronic liver failure; CS, corticosteroids; EASL, European Association for the Study of the Liver; eLT, early liver transplantation; iACLF, incident acute-on-chronic liver failure; LT, liver transplantation; MDF, Maddrey’s discriminant function; MELD, model for end-stage liver disease; MELD-Na, model for end-stage liver disease—sodium; MT, medical treatment; NIAAA, National Institute on Alcohol Abuse and Alcoholism; OFs, organ failures; pACLF, prevalent acute-on-chronic liver failure; SAH, severe alcohol-associated hepatitis.
